# Supplementary material for: Implementing Patient Falls Education in Hospitals: A Mixed-Methods Trial
Source: Healthcare (Basel). 2022 Jul 13;10(7):1298. doi: 10.3390/healthcare10071298 (PMC9316918; doi:10.3390/healthcare10071298)
Supplement: Supplementary file 1 [file healthcare-10-01298-s001.zip › healthcare-1750118-supplementary.pdf]

Supplementary File S1. Pre- and Post-intervention survey

**Pre-intervention survey**	**Strongly disagree (1)**	**Disagree (2)**	**Undecided (3)**	**Agree (4)**	**Strongly agree (5)**
1. For me, taking measures to reduce my risk of falling would be useful in hospital					
2. Most people whose opinion I value approve of me taking measures to reduce my risk of falling in hospital					
3. I feel positive about reducing my overall risk of falling in hospital					
4. I am aware of the measures needed to reduce my risk of falling in hospital					
5. I am confident that if I wanted to, I could reduce my risk of falling in hospital					
6. While I'm in hospital, I intend to take measures to reduce falls or my risk of falling					
7. I have a clear plan of how I will take measures to reduce falls or my risk of falling in hospital					

**Post-intervention survey**	**Strongly disagree (1)**	**Disagree (2)**	**Undecided (3)**	**Agree (4)**	**Strongly agree (5)**
1. For me, taking measures to reduce my risk of falling is useful in hospital					
2. Most people whose opinion I value approve of me taking measures					

to reduce my risk of falling in hospital					
3. I feel positive about reducing my overall risk of falling in hospital					
4. I am aware of the measures needed to reduce my risk of falling in hospital					
5. I am confident that if I wanted to, I could reduce my risk of falling in hospital					
6. In hospital, I used measures to reduce falls or my risk of falling					
7. I had a clear plan of how to reduce falls or my risk of falling in hospital					
8. On a scale of 1-5, how would you rate the importance of receiving face-to-face education for falls prevention in hospital					
9. What did you think about the education you received on the ward about falls prevention?					

Supplementary File S2. Interview schedule for clinical staff

EMPOWER Education Trial

Semi-Structured Interview Guide for Clinical Staff

Introduction

Preamble:

Thank you for your time today and agreeing to take part in this interview. The purpose of this interview is to obtain your views about the EMPOWER Patient Education Trial implemented here at Holmesglen Private. This includes your views about the clinician training sessions, carrying out the intervention, and the implementation process of the trial.

The focus of the trial was on empowering patients to reduce their risk of falling on the ward. I will ask you about your views on the barriers and facilitators to the implementation of the EMPOWER trial and intervention at different levels. It will be about what you perceived personally, at a ward level and at an organisational level.

As outlined in the Participant Information and Consent Form, this interview will be audio recorded. All responses are confidential and only the researchers working on this study will have access to the transcript of the interview. All data and presentation of findings will be de-identified.

[START AUDIO RECORDING HERE]

Opening questions:

1. What is your current role?

Interview questions:

PERCEPTIONS OF THE PATIENT EDUCATION INTERVENTION

2. What did you think of the EMPOWER patient education strategy?
    a. Did you carry out the EMPOWER education intervention on Kendall?
3. At a personal level, what do you think helped or did not help you implement the EMPOWER patient education trial?
    a. (Prompt) What aspects of this intervention and the trial worked for you?
    b. (Prompt) What did you feel were barriers to carrying out this intervention?
    c. (Prompt) In what way did knowing this patient education was evidence-based impact on your motivation to deliver the patient education?

PERCEPTIONS ON THE PROCESS OF IMPLEMENTING CHANGE AT A WARD LEVEL

4. Can you identify staff behaviours on the ward that may have helped or made it difficult to implement the EMPOWER patient education trial?
    a. (Prompt) What were these?
    b. (Prompt) What do you think influenced these behaviours?
5. Do you think there were any interprofessional aspects that worked well or could have worked better? (*e.g. teamwork, communication, culture)*
    a. (Prompt) How do you think these aspects could be improved?

PERCEPTIONS ON THE PROCESS OF IMPLEMENTING CHANGE AT AN ORGANISATIONAL LEVEL

6. Can you identify any organisational factors that may have facilitated or were a barrier to implementing the EMPOWER patient education trial? (e.g., *formal or informal processes, leadership, communication, culture)*
    a. (Prompt) What were these?
    b. (Prompt) What would you do differently if you were in charge of this project?

PERCEPTIONS OF THE TRAINING SESSIONS FOR THE EMPOWER TRIAL

7. Were you able to participate in the clinician education for the EMPOWER trial? *(If yes, continue on to the next questions)*
    a. What aspects of this training assisted you to implement the patient education?
    b. After the staff education, did you feel confident and prepared to deliver the intervention to your patients? (Prompt) If no, why?
    c. How could the education be improved? (Prompt) What did you feel about the amount of time made available for the education?
    d. Did you appreciate how the new patient education program could help to increase safety or reduce falls in your clinical area?
8. If you were going to educate other staff about this intervention, how would you do it to ensure engagement and commitment?

Supplementary File S3. Interview schedule for managers

EMPOWER Education Trial

Semi-Structured Interview Guide for Managers

Introduction

Preamble:

Thank you for your time today and agreeing to take part in this interview. The purpose of this interview is to obtain your views about the EMPOWER Patient Education Trial here at Holmesglen Private.

The focus of the trial was on empowering patients to reduce their risk of falling on the ward. I will ask you about your views on the barriers and facilitators to the implementation of the EMPOWER trial at different levels. It will be about what you perceived personally, at a ward level and at an organisational level.

As outlined in the Participant Information and Consent Form, this interview will be audio recorded. All responses are confidential and only the researchers working on this study will have access to the transcript of the interview. All data and presentation of findings will be de-identified.

[START AUDIO RECORDING HERE]

Opening questions:

9. What is your current role?

Interview questions:

PERCEPTIONS ON THE EMPOWER TRIAL AND INTERVENTION IMPLEMENTATION PROCESS

10. At a personal level, what do you think helped or did not help you implement the EMPOWER patient education trial?
    a. (Prompt) What aspects of this trial worked for you?
    b. (Prompt) What did you feel were barriers to implementing this trial?

PERCEPTIONS ON THE PROCESS OF IMPLEMENTING CHANGE AT A WARD LEVEL

11. Can you identify staff behaviours that may have helped or made it difficult to implement the EMPOWER patient education trial?
    a. (Prompt) What were these?
    b. (Prompt) What do you think influenced these behaviours?
12. Do you think there were any interprofessional aspects that worked well or could have worked better? (*e.g. teamwork, communication, culture)*
    a. (Prompt) How do you think these aspects could be improved?

PERCEPTIONS ON THE PROCESS OF IMPLEMENTING CHANGE AT AN ORGANISATIONAL LEVEL

13. Can you identify any organisational factors that may have facilitated or were a barrier to implementing the EMPOWER patient education trial? (e.g., *formal or informal processes, leadership, communication, culture)*
    a. (Prompt) What were these?
    b. (Prompt) What would you do differently if you were in charge of this project?

Supplementary File S4. Participant quotes

THEMES	POINTS RAISED	QUOTES
**Barriers**	Patient status affected patient engagement: Cognitive impairment, reduced medical status, and reduced insight	"…the biggest barrier was probably the cognitive impaired patients, anyone with a language barrier" (P5, enrolled nurse)<br><br>"…the setting…being medical, we can [see]…a lot of very confused patients…So, it became difficult to deliver the education because…we had a lot of patients who probably weren't appropriate, or just cognitively weren't going to get the most out of that sort of delivery of falls education" P3
	Staff attitudes reduced engagement with implementation	"…if it's more of a chronic problem and previous attempts have not been so successful, then sometimes there can be an element of scepticism…So previous failures might lead into that." (P1, physiotherapist)<br><br>"I suppose there was a bit of, 'We already do this,' you know, 'This is just even more work for us,'" (P5, enrolled nurse)
	Lack of staff input into designing the intervention and processes	"…there are directives that come down from the top that are made without any, what feels like, without much input from us on the bottom line" (P7, enrolled nurse)
	Use of unrealistic scenarios during training	"Where, in a realistic, real-life work situation where you have five patients to look after, this one is buzzing, maybe this one need toileting… In the middle of an education program someone can buzz, … there can be an emergency call in another room, and the alarms are going off. You need to attend to that. You need to end your education pathway to [work through it]. So, yeah, it wasn't really practical in the real-life situation." (P2, registered nurse)
	Lack of emphasis on intra and interprofessional collaboration during the trial	"…overall on the ward, there might be – possibly like there might not be the best sort of communication across all the other disciplines, and that's where you might run into trouble." (P1, physiotherapist)

		"I think it didn't necessarily strengthen or change the relationship, or the interactions that the team had. I think if anything it was just that we maybe had them as slightly different [way of interaction]." (P3, enrolled nurse)
	Time constraints and heavy workloads	"How long it took to have the initial conversation was a barrier. That's not a fault of the study, I don't think, I think it speaks to how busy we are as nurses, and I mean, …we have five patients, and if at oftentimes we have patients who are cognitively confused or just really heavy – we get a lot of full nursing care patients on the ward. So, finding the extra time to deliver the education in [steps], it's a little bit impossible some days." (P3, enrolled nurse)
	Limited leadership support and organisational management	"There wasn't any consultation with the staff of how would be the best way to deliver this, and no real conversation regarding what were the expectations of the staff going into this." (P3, enrolled nurse)
	Limitations in current processes to support intervention	"…the other thing that we could have probably helped more with it is, what was the baseline of what was actually going on, current practice? And were we aware of the exact current practice of that particular ward?" (P6, executive)<br><br>"We know that during this period, from what I recall, the manager…had some leave…There was some probably not-ideal communication there, we could have probably tightened up on that. And so, there was some areas where our communication probably wasn't as tight as it should have been, and probably some other issues going on there, personal issues." (P6, executive)
**Enablers**	Fostering patient empowerment and engagement with falls prevention	"…positives was getting the patient involved in their aspect of their care." (P1, physiotherapist)<br><br>"…it gave me a little bit of a different perspective on how to look at falls education…it encouraged us as clinicians to…put a bit more emphasis on the patient identifying their own falls risk, which…is a good

		strategy in terms of creating an environment where the patient is involved in keeping themselves safe." (P3, enrolled nurse)
	Building on existing strengths of intra and interprofessional collaboration	"I didn't have a lot of discussions about this program, but allied health is always discussing with us nurses about the mobility and falls risk and so forth of the patient." (P5, enrolled nurse)
	Consistently reinforcing falls education across the healthcare team	"…we were all educated about it, around about the same time, so we…were on the same page, which I think was more or less a protective factor in terms of implementing the work" (P3, enrolled nurse)
	Using a structured and embedded approach to falls prevention	"It wasn't absolutely new to be fair, but I think it's the fact that we were doing it in a formalised way across…all staffing levels, and that obviously created a different level of awareness." (P1, physiotherapist)
	Modelling scenarios during training	"…modelling empowering patients through education and telling them about falls risk…was helpful. Just the approach was helpful…that helped because we saw them doing it, saw them saying the things that they thought was important." (P2, registered nurse)
	Using an evidence-based intervention was motivating	"…you're not just doing because [it's] one of those things that you have to do in your shifts. You're doing it because it's evidence backing what you're doing, [it's] evidence that suggests that doing this actually helps the patient from falling and the cycle of events that happen after the fall." (P2, registered nurse)
	Having support from senior staff	"I think the organisation was quite supportive of the Empower programs. The ANUMs…and the NUMs in our ward, they would tell us, "This folder here is for the Empower program. Make sure you do it…" They'll say things like that." (P2, registered nurse)
**Recommended strategies**	Leadership engagement and support with stakeholder input	"…clearly, once we've got the buy-in from the executive, and then there needs to be the buy-in from the manager to be on the same page, in terms of what we're trying to achieve and what the actual issues are, and the involvement in the actual research project, and to really have that buy-in, that we need to give this a go. And then, of course, then it's disseminated amongst the ANUMs and then filtered down to the other staff." (P6, executive)
	Changes in systems and policies to support implementation	"…the importance of having some structure and governance and your standard agenda items and the meetings that we participate in together…they do keep you aligned and on track." (P6, executive)

	Further focus on intra and interprofessional collaboration	"They don't follow-up like, 'Did you get a chance to do this?' 'How did the patient take it?' So, I feel like if there was more follow-up from the ANUMs doing the shift…" (P2, registered nurse)<br><br>"…it also helps too just to sort of stop that kind of – that us and them sort of factor. If…the speech pathologist walks in and sees a patient and walks out again…and just kind of says, 'I've finished with them now. You can go in and sort them out', it creates a very us and them between the allied and the nursing staff." (P7, enrolled nurse)
	Engaging staff and providing support	"…if we are trying to change practice, or we are trying to embed something…we do need to give it the attention and that time, but at the same time, make it easy for them to see that it's important and part of their whole everyday intervention or practice." (P6, executive)
	Embedding into usual practice across a continuum of care	"I've always been encouraging of my nurses to make few things as part of admission. We have some standard protocols that needs to be done while the patient is getting admitted, so just as part of the admission process I think that might make a habit of them to just do it" (P4, assistant nurse unit manager)
	Accountability among staff	"…checking in on your staff. What are you doing at your handovers, your bedside handovers, your huddles? And all the other things that you do, around what is the focus for the month. And it could be falls." (P6, executive)<br><br>"…it's about knowing your patient and being accountable you start to know your patient. And then if you know your patient, then you start to sort of go to that next step and think about ways to improve their care." (P1, physiotherapist)
